# Overexpression of apoptosis inducing factor aggravates hypoxic-ischemic brain injury in neonatal mice

**DOI:** 10.1038/s41419-020-2280-z

**Published:** 2020-01-30

**Authors:** Tao Li, Kenan Li, Shan Zhang, Yafeng Wang, Yiran Xu, Shane J. F. Cronin, Yanyan Sun, Yaodong Zhang, Cuicui Xie, Juan Rodriguez, Kai Zhou, Henrik Hagberg, Carina Mallard, Xiaoyang Wang, Josef M. Penninger, Guido Kroemer, Klas Blomgren, Changlian Zhu

**Affiliations:** 1 Henan Key Laboratory of Child Brain Injury, Institute of Neuroscience and Third Affiliated Hospital, Zhengzhou University, Zhengzhou, 450052 China; 2 Center for Brain Repair and Rehabilitation, Institute of Neuroscience and Physiology, Sahlgrenska Academy, University of Gothenburg, Gothenburg, 40530 Sweden; 3 Department of Pediatrics, Children’s Hospital Affiliated of Zhengzhou University, Zhengzhou, 450018 China; 4 Institute of Molecular Biotechnology, Austrian Academy of Sciences, 1030 Vienna, Austria; 5 Department of Women’s and Children’s Health, Karolinska Institutet, Stockholm, Sweden; 6 Centre of Perinatal Medicine and Health, Sahlgrenska Academy, University of Gothenburg, Gothenburg, 40530 Sweden; 7 Institute of Neuroscience and Physiology, Sahlgrenska Academy, University of Gothenburg, Gothenburg, 40530 Sweden; 8 Department of Medical Genetics, Life Sciences Institute, University of British Columbia, Vancouver, Canada; 9 Equipe labellisée par la Ligue contre le cancer, Université Paris Descartes, Université Sorbonne Paris Cité, Université Paris Diderot, Sorbonne Université, INSERM U1138, Centre de Recherche des Cordeliers, Paris, France; 10 Metabolomics and Cell Biology Platforms, Institut Gustave Roussy, Villejuif, France; 11 Pôle de Biologie, Hôpital Européen Georges Pompidou, AP-HP, Paris, France; 12 Suzhou Institute for Systems Biology, Chinese Academy of Sciences, Suzhou, China; 13 Karolinska Institute, Department of Women’s and Children’s Health, Karolinska University Hospital, Stockholm, Sweden; 14 Pediatric Hematology and Oncology, Karolinska University Hospital, Stockholm, Sweden

**Keywords:** Cell death in the nervous system, Developmental disorders

## Abstract

Apoptosis inducing factor (AIF) has been shown to be a major contributor to neuron loss in the immature brain after hypoxia-ischemia (HI). Indeed, mice bearing a hypomorphic mutation causing reduced AIF expression are protected against neonatal HI. To further investigate the possible molecular mechanisms of this neuroprotection, we generated an AIF knock-in mouse by introduction of a latent transgene coding for flagged AIF protein into the Rosa26 locus, followed by its conditional activation by a ubiquitously expressed Cre recombinase. Such AIF transgenic mice overexpress the pro-apoptotic splice variant of AIF (AIF1) at both the mRNA (5.9 times higher) and protein level (2.4 times higher), but not the brain-specific AIF splice-isoform (AIF2). Excessive AIF did not have any apparent effects on the phenotype or physiological functions of the mice. However, brain injury (both gray and white matter) after neonatal HI was exacerbated in mice overexpressing AIF, coupled to enhanced translocation of mitochondrial AIF to the nucleus as well as enhanced caspase-3 activation in some brain regions, as indicated by immunohistochemistry. Altogether, these findings corroborate earlier studies demonstrating that AIF plays a causal role in neonatal HI brain injury.

## Introduction

Hypoxic-ischemic encephalopathy (HIE) is a severe central nervous system injury that manifests in neonates as a consequence of perinatal asphyxia. HIE is an important cause of neonatal mortality and of serious and devastating lifelong disabilities such as cerebral palsy, neurosensory deficits, and cognitive impairments in both term and preterm neonates^1^. The incidence of HIE ranges from 1 to 8 per 1000 live births in developed countries and is as high as 26 per 1000 live births in underdeveloped countries^1^. HIE accounts for up to 22% of all neonatal deaths worldwide^2^. Therapeutic hypothermia has been widely implemented and modestly improves outcome in full-term infants^3,4^. Erythropoietin treatment also demonstrates remarkable neuroprotection in both term and preterm infants^5–7^. However, hypothermia therapy is limited to full-term infants, and the window of opportunity for erythropoietin treatment is still unclear. Thus, a better understanding of the mechanisms of neuronal cell death and brain injury after HI is warranted in order to develop novel strategies for the prevention and treatment of neonatal brain injury.

The neonatal brain is particularly vulnerable to oxidative damage because of an elevated rate of oxygen consumption, low concentrations of antioxidants, and the availability of redox-active iron^8^. Therefore, oxidative stress, excitotoxicity, inflammatory responses, and activation of several distinct cell death pathways, including apoptosis, necrosis, necroptosis, ferroptosis, and autophagy, commonly occur in the neonatal brain after HI^9–12^. Because apoptosis is critical for normal brain development and determines the size and shape of the central nervous system^10^, the neonatal brain is likely more susceptible to this cell death pathway than the adult brain^13^. Thus, apoptosis is thought to account for a significant portion of the neuronal cell loss associated with neonatal HI^14^.

AIF is a flavoprotein located in the mitochondrial intermembrane space and has a dual role in controlling cell survival and death^15,16^. Since its discovery, the role of AIF in the apoptotic process has been studied extensively^17,18^. In response to pro-apoptotic signals, AIF translocates from the mitochondria to the nucleus where it interacts with DNA and stimulates chromatin condensation as well as high molecular weight DNA fragmentation^19^. AIF is synthesized in the cytosol and subsequently imported into the mitochondrial intermembrane space where it is required for maintaining mitochondrial morphology and cristae structure^20^. Mitochondrial morphology is dynamically controlled by a balance between organelle fission and fusion^21^, and this dynamic equilibrium can be disrupted by AIF deficiency^22^. Studies show that mitochondrial dynamics can be disrupted by HI injury in the immature brain and by ischemia/reperfusion in the adult brain^18,23–27^. Thus, mitochondrial dynamics might be related to the pathophysiological processes of HI-induced brain injury. Our previous studies have shown that AIF is a major contributor to neuronal loss induced by neonatal cerebral HI^17,28^. Harlequin mice, which bear a hypomorphic mutation in the *Aif* gene causing ~80% reduction in AIF protein level, experience significant neuroprotection in ischemic brain injury models^17,29^. However, it has been debated whether it is the reduction of AIF translocation to the nucleus or the downregulation of mitochondrial respiratory activity that accounts for reduced neuronal cell death in Harlequin mice^14,17,29^. Indeed, AIF deficiency entails a defect in oxidative phosphorylation that, on theoretical grounds, could reduce the production of deleterious reactive oxygen species (ROS) in the context of HI^30^. To further confirm the effect of AIF on neuronal cell death, we generated an AIF knock-in mouse by introduction of a latent transgene (inactivated by a Lox-Stop-Lox cassette) coding for flagged AIF protein into the Rosa26 locus, followed by its conditional activation by Cre recombinase expressed under the control of the ubiquitous *Actin* promoter. By using AIF-overexpressing transgenic mice, we investigated the effect of AIF on neonatal brain injury after HI and potential molecular mechanisms. We found that AIF overexpression aggravated neonatal brain injury after HI.

## Results

### AIF-overexpressing mice have a normal phenotype

**Fig. 1:**
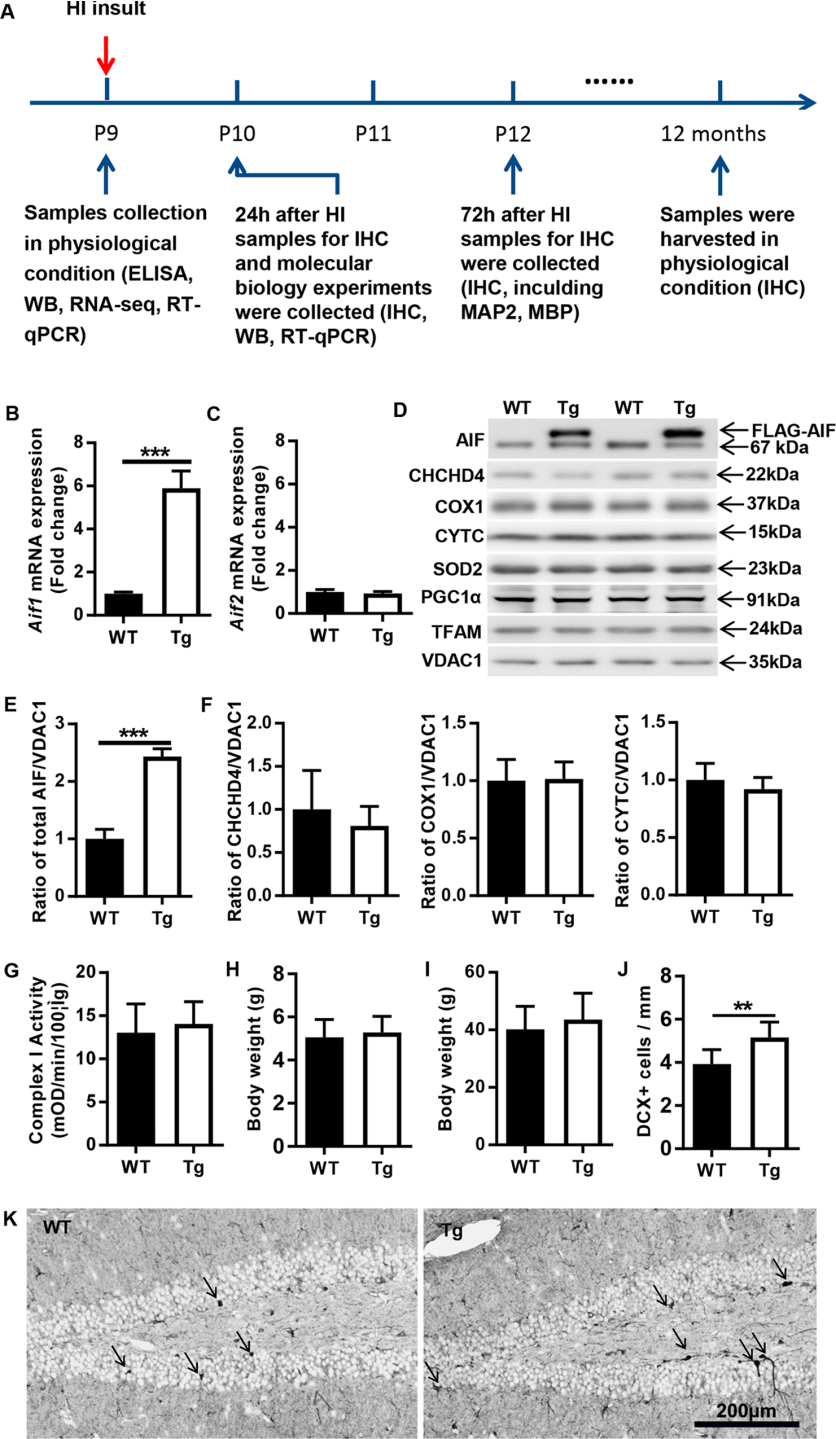
Expression of mitochondria-related proteins in P9 AIF Tg mice under physiological conditions. **a** The timeline for the entire study, including sample collection time points and the experiments that were performed at different time points. **b** *Aif1* mRNA expression was upregulated in AIF Tg mice compared to WT mice at P9 under physiological conditions (5.89 ± 0.80 vs. 1.00 ± 0.08, *n* = 6/group, ***p *<* 0.001). **c** *Aif2* mRNA expression was not significantly different between WT and AIF Tg mice at P9 (1.00 ± 0.11 vs. 0.93 ± 0.09, *n* = 6/group, *p* = 0.227). **d** Representative immunoblots of the mitochondrial fraction from cortical tissue of P9 WT and AIF Tg mice. A greater amount of exogenous AIF (upper band of AIF) than endogenous AIF (lower band of AIF) was seen in the AIF Tg mice. **e** Total AIF protein expression in AIF Tg mice (two bands) was dramatically increased compared to WT mice (one band) (2.44 ± 0.13 vs. 1.00 ± 0.17, respectively, *n* = 6/group, ***p < 0.001), but there was no significant difference in endogenous AIF expression (1.08 ± 0.14 vs. 1.00 ± 0.31, respectively, *n* = 6/group, *p* = 0.5577). **f** Semi-quantification of some mitochondria-related proteins (CHCHD4, COX1, CYTC, and SOD2) did not show any significant differences between WT and AIF Tg mice under physiological conditions (*n* = 6/group). **g** OXPHOS complex I activity was determined in the mitochondrial fraction of cortical tissue of P9 WT and AIF Tg mice, and no significant difference was found (13.04 ± 3.32 vs. 14.05 ± 2.58, *n* = 6/group, *p* = 0.5677). **h** The body weight of P9 mouse pups was not significantly different between WT and AIF Tg mice (5.06 ± 0.82 g vs. 5.28 ± 0.74 g, respectively, *n* = 54/group, *p* = 0.1407). **i** The body weight at one year of age was not significantly different between WT and AIF Tg mice (40.18 ± 8.04 g vs. 43.61 ± 9.13 g, respectively, *n* = 8 in WT mice, *n* = 13 in AIF Tg mice, *p* = 0.393). **j** The DCX-positive cell density in the granular layer of the dentate gyrus of AIF Tg mice was significantly higher than WT mice at one year of age (3.93 ± 0.66 cells/mm vs. 5.15 ± 0.71 cells/mm, *n* = 8 in WT mice, *n* = 6 in AIF Tg mice, **p < 0.01). **k** Representative pictures of DCX staining in dentate gyrus regions in WT and AIF Tg mice at one year of age. Black arrows indicate the positive cells.

Two isoforms of AIF have been identified according to whether exon 2a or 2b of the *Aif* gene is expressed. AIF1 (which uses exon 2a) was the first to be described and is the most abundant and ubiquitous isoform, while AIF2 (which uses exon 2b) is restricted to the central nervous system and thus is called the brain-specific isoform^31^. AIF2 is more strongly anchored to the inner mitochondrial membrane than AIF1, and we previously showed that a lack of AIF2 aggravated cerebral damage in a model of neonatal HI^32^. In contrast, reduction of AIF1 expression reduces neuronal cell loss under identical conditions^17^. Driven by these considerations, we first investigated which AIF isoform would be overexpressed in transgenic mice. The relative abundance of *Aif1* and *Aif2* mRNA transcripts in the brains of WT and AIF Tg mice at postnatal day (P) 9 was determined by quantitative reverse transcription PCR (RT-qPCR). Under physiological conditions, *Aif1* mRNA expression in AIF Tg mice was 5.9 times higher than in the WT mice at P9 (Fig. 1b). In contrast, there was no significant difference in *Aif2* mRNA expression between WT and AIF Tg mice (Fig. 1c). Compared with WT controls, two AIF protein bands were detected in the AIF Tg mice, the upper band was the transgenic AIF with a FLAG tag (FLAG-AIF), which has a higher molecular weight and so can be distinguished from endogenous AIF, which was represented by the lower band. (Fig. 1d). The abundance of total AIF protein was 2.4 times greater in AIF Tg than in WT mice at P9 (Fig. 1e). Other mitochondria-related proteins, including coiled-coil-helix-coiled-coil-helix domain-containing protein 4 (CHCHD4), cytochrome C oxidase subunit I (COX1), cytochrome C (CYTC), a peripheral protein of the mitochondrial inner membrane (which functions as an essential electron shuttle between complex III and complex IV of the respiratory chain, but also plays a prominent role in post-mitochondrial caspase activation), superoxide dismutase 2 (SOD2), voltage dependent anion channel 1 (VDAC1), and mitochondrial biogenesis-related proteins, including peroxisome proliferator-activated receptor gamma coactivator 1-alpha (PGC1α) and transcription factor A, mitochondrial (TFAM), showed no difference between WT and AIF Tg mice at P9 (Fig. 1d, f). To further evaluate oxidative phosphorylation in the brain of AIF Tg mice, the activity of mitochondrial oxidative phosphorylation (OXPHOS) complex I was measured. This activity exhibited a trend towards a non-significant increase in AIF Tg mice (Fig. 1g). AIF Tg mice normally survived well beyond 1 year of age without showing any major phenotypic or behavioral alterations. No significant difference in body weight was detected in P9 or 12-month-old WT and AIF Tg mice (Fig. 1h, i). The neuronal proliferation marker doublecortin (DCX), which is a microtubule-associated protein expressed by neuronal precursor cells, was determined in 1-year-old mice. The density of DCX-positive cells in the granular layer of the dentate gyrus was higher in AIF Tg mice than in WT mice (*p* = 0.006) (Fig. 1j, k).

**Fig. 2:**
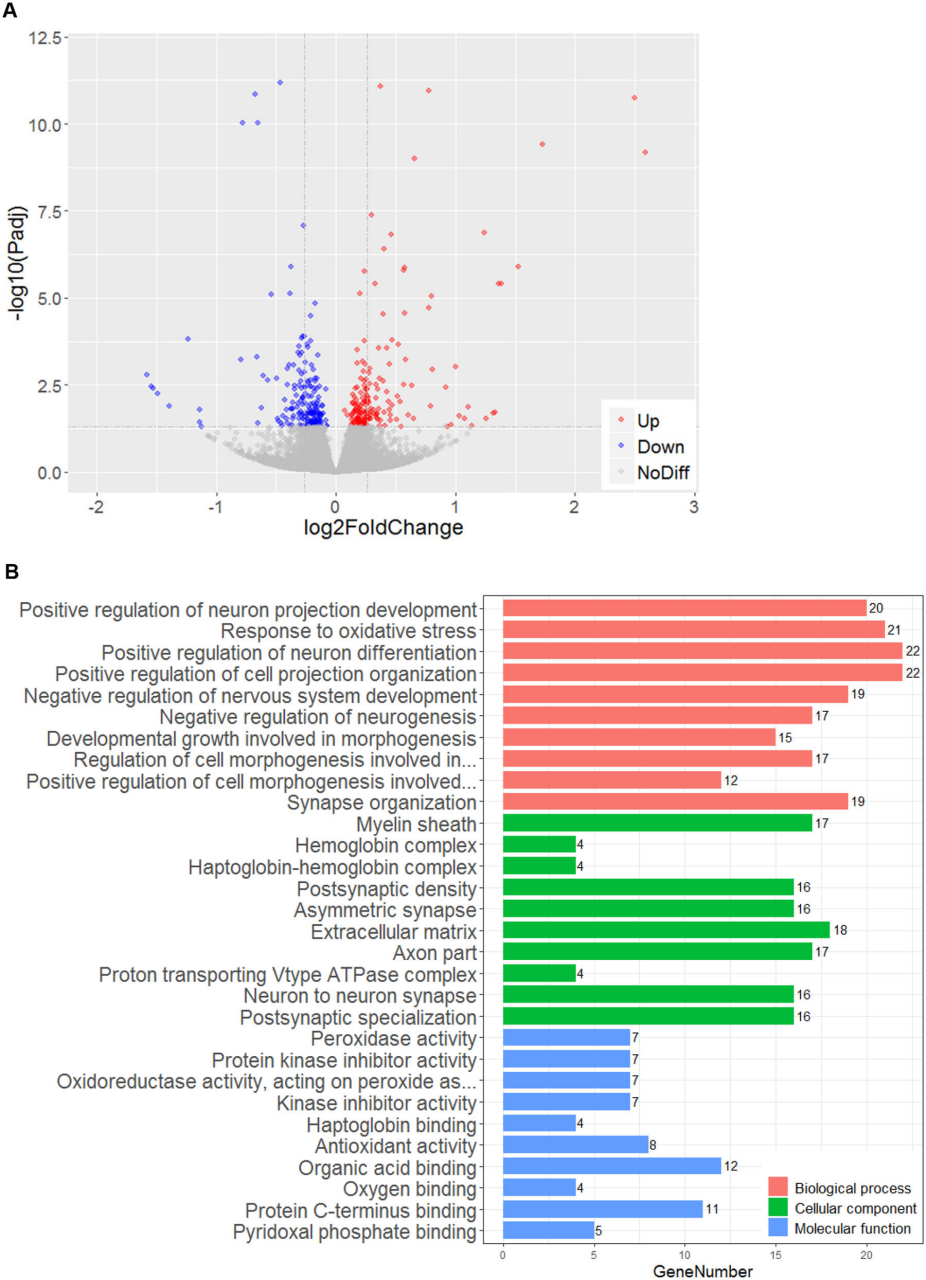
AIF overexpression had no significant impact on the transcriptome in the cortex of P9 mice. **a** Volcano plot showing DEGs between WT male mice and AIF Tg male mice (*n* = 6/group). The negative log10-transformed adjusted *p*-values (Padj) test the null hypothesis of no difference in expression levels between WT and AIF Tg male mice (*Y*-axis) and are plotted against the average log2 fold changes in the expression (*X*-axis). Non-significantly expressed genes are plotted in grey (*p* < 0.05 was used for the filter and is indicated by the dashed line parallel with *X*-axis). Significantly upregulated and downregulated genes are plotted in red and blue, respectively (369 out of 21,762 genes in total, with 183 genes upregulated and 186 genes downregulated). The two dashed lines parallel with the *Y*-axis indicate an absolute log2 fold change equal to 0.2. **b** Top ten classified GO terms in three ontologies. GO classification was performed based on the DEGs (369 genes in total). The *X*-axis represents the number of DEGs, and the *Y*-axis represents the GO terms.

To further investigate whether AIF overexpression has an impact on the mRNA transcriptome under physiological conditions, the transcriptomes of six P9 male mouse brain tissues from the cortex were determined by RNA sequencing. Even when using the relaxed criterion of *p* < 0.05, the data analysis showed that only 369 of the total of 21,762 genes were differentially expressed in AIF Tg mice compared to WT mice (Fig. 2a). Among these 369 genes, 183 were upregulated and 186 were downregulated. Gene ontology (GO) term classification was performed on the differentially expressed genes (DEGs) in three ontologies (molecular biological function, cellular component, and biological process), but no specific terms were found among the top ten classified GO terms (Fig. 2b).

### AIF overexpression aggravates brain injury in neonatal HI mice

**Fig. 3:**
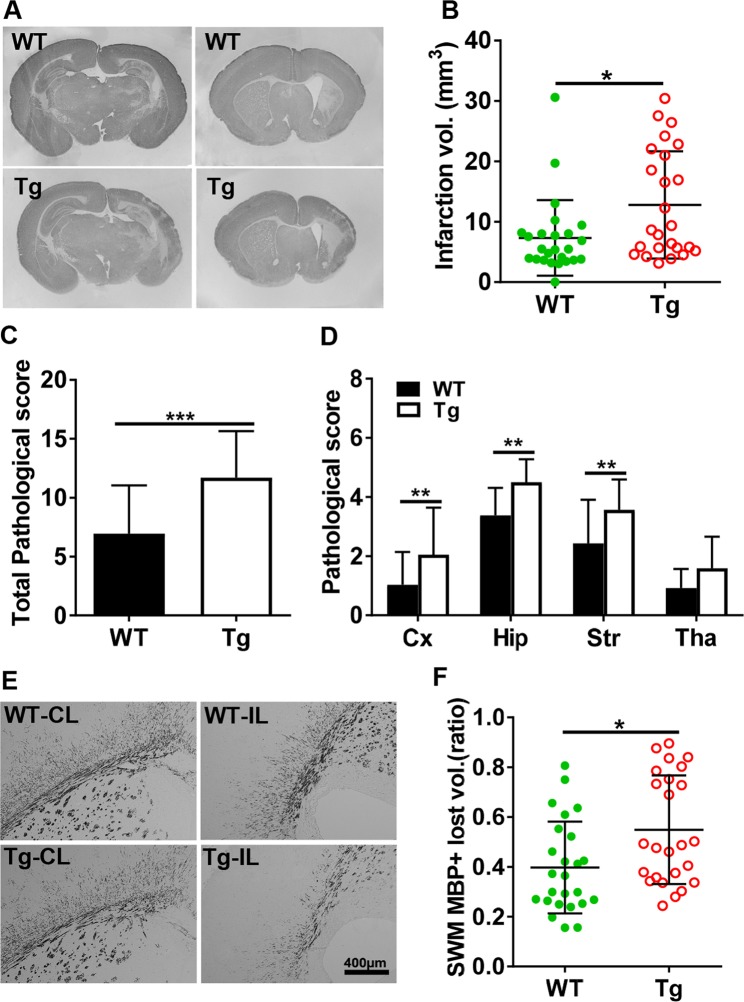
AIF overexpression increased brain injury after HI. **a** Representative MAP2 staining of coronal brain sections 72 h after HI at the levels of the dorsal hippocampus (left panels) and striatum (right panels) from WT and AIF Tg mice. **b** The infarction volume was measured at 72 h after HI in WT and AIF Tg mice (7.32 ± 6.25 mm^3^ vs. 12.78 ± 8.89 mm^3^, respectively, *n* = 25/group, including 14 males and 11 females. *p *<* 0.05). **c** The total pathological score was evaluated at 72 h after HI in WT and AIF Tg mice (6.96 ± 4.10 vs. 11.70 ± 3.95, respectively, *n* = 25/group, ***p < 0.001). **d** The pathological scores were evaluated in different brain regions in WT and AIF Tg mice, including the cortex (Cx, 1.03 ± 1.11 vs. 2.05 ± 1.59, respectively, **p < 0.01), hippocampus (Hip, 3.38 ± 0.93 vs. 4.50 ± 0.77, respectively, **p < 0.01), striatum (Str, 2.43 ± 1.48 vs. 3.56 ± 1.03, respectively, **p < 0.01), and thalamus (Tha, 0.92 ± 0.64 vs. 1.59 ± 1.07, respectively, *p* = 0.1356). **e** Representative MBP staining of coronal brain sections revealed the myelin structure in the subcortical white matter of the CL and IL hemispheres at 72 h after HI. **f** Quantification of the tissue loss ratio in the subcortical white matter (SWM) showed more white matter loss in AIF Tg mice than in WT mice at 72 h after HI (0.55 ± 0.22 vs. 0.40 ± 0.18, respectively, *n* = 25/group, including 14 males and 11 females. *p *<* 0.05).

HI-induced brain injury in neonatal mice was evaluated via immunohistochemistry staining for markers of gray matter (MAP2, microtubule associated protein 2) and white matter (MBP, myelin basic protein) in brain tissues at 72 h after HI. The injury encompassed the cortex, hippocampus, striatum, and thalamus, as indicated by MAP2 staining of brain coronal sections, and AIF overexpression significantly increased the severity of brain injury after HI (Fig. 3a). The extent of gray-matter injury, as indicated by the infarction volume, increased by 74.6%, from 7.32 ± 6.25 mm^3^ in WT mice to 12.78 ± 8.89 mm^3^ in AIF Tg mice (*p* = 0.0154) (Fig. 3b). No significant difference was found between male and female AIF Tg mice (13.62 ± 9.30 mm^3^ vs. 11.71 ± 8.66 mm^3^, respectively, *p* = 0.7907). The total neuropathological score was significantly higher for AIF Tg mice compared to WT controls (*p* < 0.001) (Fig. 3c). AIF overexpression increased HI-induced brain injury in all observed brain regions, in particular in the cortex, hippocampus, and striatum (Fig. 3d). Myelination was visualized in the subcortical white matter by MBP staining at 72 h after HI (Fig. 3e), and the total lost volume of subcortical white matter in the ipsilateral hemisphere was greater in the AIF Tg mice as compared to WT mice at 72 h after HI (*p* = 0.0107) (Fig. 3f). However, no difference was found between male and female in AIF Tg mice.

### AIF overexpression affects apoptotic cell death pathways

**Fig. 4:**
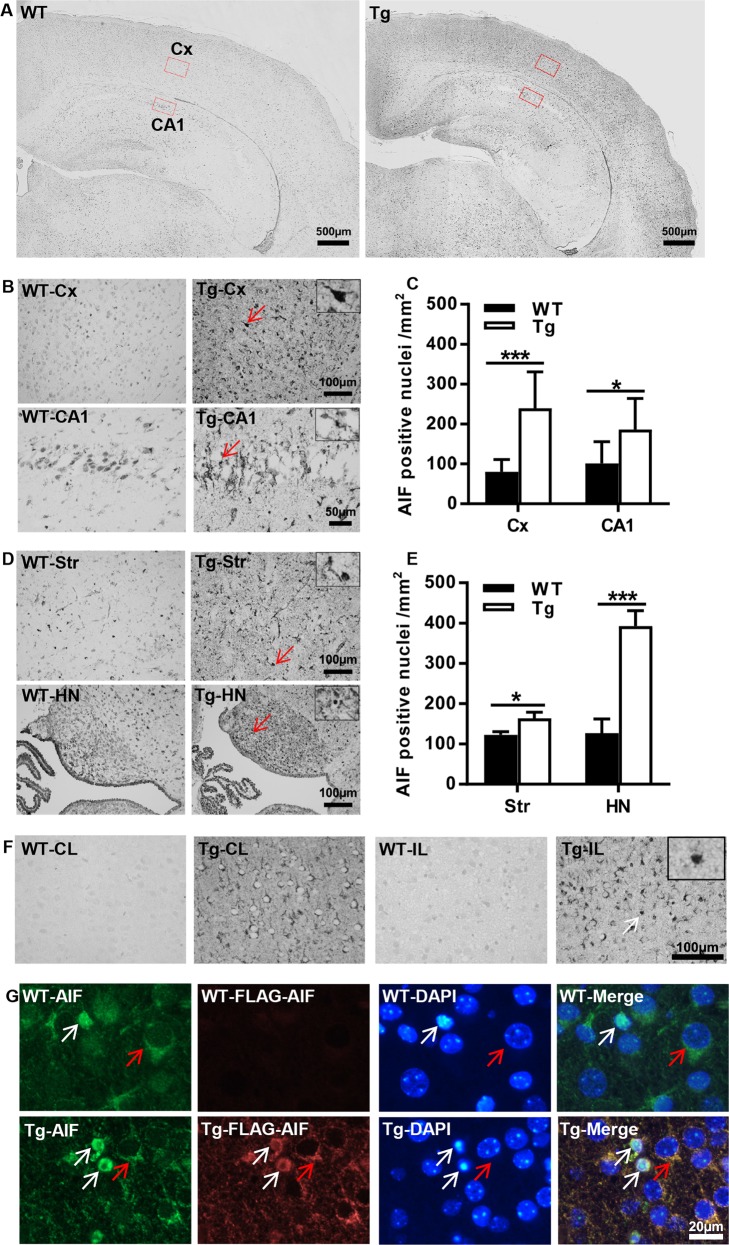
AIF overexpression increased AIF nuclear translocation in the brain after HI. **a** Representative panoramic images of AIF staining showing the cortex and hippocampus in WT and AIF Tg mice at 24 h post-HI. **b** Higher magnifications of AIF staining images show AIF-positive nuclei in the cortex (Cx) area (upper panels) and hippocampal cornus ammonis 1 (CA1) area (lower panels) in WT and AIF Tg mice at 24 h post-HI. **c** Quantification of AIF-positive nuclei in WT and AIF Tg mice at 24 h after HI in the cortex (80.4 ± 30.7 cells/mm^2^ vs. 239.2 ± 91.8 cells/mm^2^, 95% confidence interval (CI) 78.5–239.1, respectively, *n* = 8/group, ***p < 0.001) and CA1 (101.1 ± 54.6 cells/mm^2^ vs. 186.1 ± 78.4 cells/mm^2^, 95% CI 4.6–165.3, respectively, *n* = 8/group, *p < 0.05). **d** Higher magnifications of AIF staining images showing the AIF-positive nuclei in the striatum (Str) (upper panels) and habenular nuclei (HN) (lower panels) in WT and AIF Tg mice at 24 h post-HI. **e** Quantification of AIF-positive nuclei in in WT and AIF Tg mice at 24 h after HI in the striatum (122.8 ± 21.8 cells/mm^2^ vs. 163.7 ± 42.6 cells/mm^2^, 95% CI 49.9–131.8, respectively, *n* = 8/group, *p < 0.05) and habenular nuclei (126.6 ± 100.0 cells/mm^2^ vs. 392.7 ± 106.6 cells/mm^2^, 95% CI 175.3–357.0, respectively, *n* = 8/group, ***p < 0.001). **f** FLAG staining in the cortex showing the expression of exogenous AIF in Tg mice, but not in WT mice (left panels, WT-CL and Tg-CL), and the nuclear translocation of exogenous AIF (right panels, WT-IL and Tg-IL). **g** Immunofluorescence staining of AIF (green) and FLAG (red) showing the nuclear translocation of both endogenous AIF and exogenous AIF at 24 h after HI (white arrows point to the injured cells, red arrows point to the normal cells).

The enhanced propensity of immature brain cells to undergo apoptosis compared to the adult brain involves both caspase-dependent and caspase-independent pathways. Thus, combined inhibition of both pathways provides synergistic protection against neonatal HI brain injury^17^. AIF is one of the major components of the caspase-independent apoptotic cell death pathway, and AIF nuclear translocation was significantly increased in several brain regions at 24 h post-HI (Fig. 4a). AIF staining showed that the number of AIF-positive nuclei in the AIF Tg mice was 3 times greater in the cortex (*p* < 0.001), 1.8 times higher in the cornu ammonis area 1 (CA1) (*p* = 0.0368), 1.3 times higher in the striatum (*p* = 0.0299), and 3.1 times higher in the habenular nuclei (*p* < 0.001) than in WT mice (Fig. 4b–e). To clarify the nuclear translocation of exogenous AIF, immunostaining with a FLAG antibody, which can recognize the transgene-encoded AIF protein (which was tagged with a FLAG^®^ peptide sequence), was performed (Fig. 4f). Immunofluorescence staining using AIF (green)/FLAG (red) antibodies confirmed that exogenous AIF contributes to HI-induced nuclear translocation and apoptotic cell death (Fig. 4g).

**Fig. 5:**
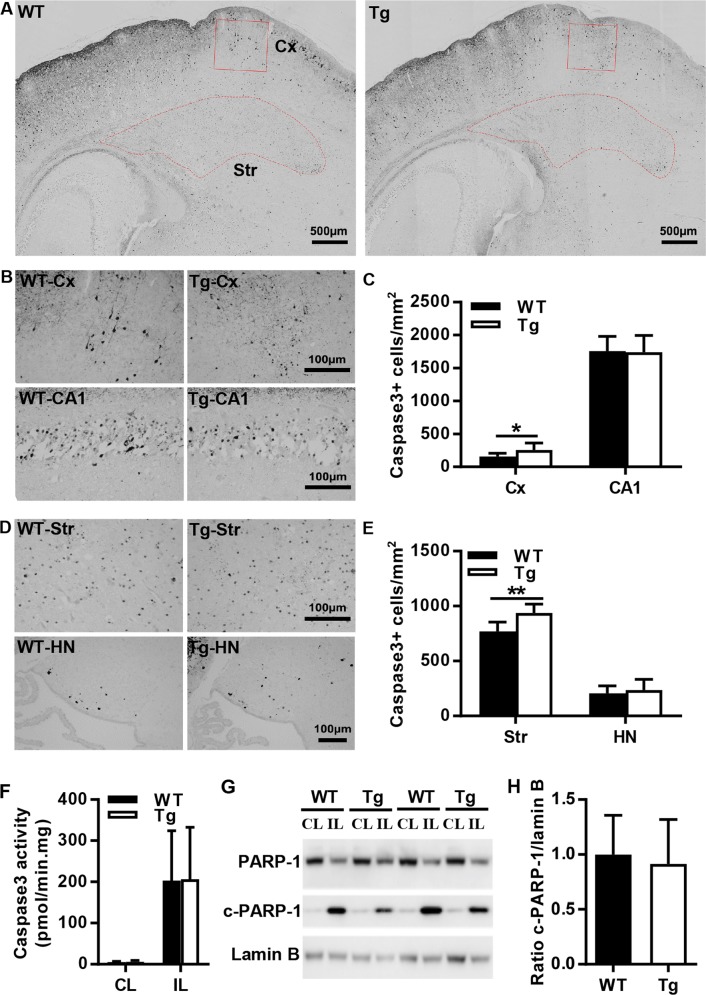
AIF overexpression increased active caspase-3–positive cells in parts of the mouse brain after HI. **a** Representative active caspase-3 staining in the cortex and striatum regions in WT and AIF Tg mice at 24 h post-HI. **b** Higher magnification of active caspase-3 staining images showing the active caspase-3-positive cells in the cortex (Cx) (upper panels) and hippocampal CA1 area (lower panels) in WT and AIF Tg mice at 24 h post-HI. **c** Quantification of active caspase-3-positive cells in WT and AIF Tg mice at 24 h after HI in the cortex (156.1 ± 50.6 cells/mm^2^ vs. 254.3 ± 106.7 cells/mm^2^, 95% CI 1.0–195.4, respectively, *n* = 7/group, *p < 0.05) and CA1 (1758.3 ± 219.5 cells/mm^2^ vs. 1,739.8 ± 252.7 cells/mm^2^, 95% CI −272.3–235.3, respectively, *n* = 8/group) area. **d** Higher magnification of active caspase-3 staining showing the active caspase-3-positive cells in the striatum (Str) (upper panels) and habenular nuclei (HN) (lower panels) in WT and AIF Tg mice at 24 h post-HI. **e** Quantification of active caspase-3-positive cells in WT and AIF Tg mice at 24 h after HI in the striatum (770.4 ± 85.5 cells/mm^2^ vs. 938.1 ± 80.3 cells/mm^2^, 95% CI 78.8–256.7, respectively, *n* = 8/group, **p < 0.01) and habenular nuclei (205.7 ± 69.6 cells/mm^2^ vs. 235.5 ± 97.9 cells/mm^2^, 95% CI −61.4–120.9, respectively, *n* = 8/group). **f** The caspase-3 activity in the cortical tissue was measured at 24 h after HI. The activity was increased dramatically in the IL hemisphere, but there was no significant difference between WT and AIF Tg mice (*n* = 6/group). **g** Immunoblotting of PARP-1 and cleaved PARP-1 in the nuclear fraction from the cortical tissue of the CL and IL hemispheres at 24 h after HI in WT and AIF Tg mice. Lamin B was used as the loading control. **h** Quantification of PARP-1 and cleaved PARP-1 did not show any significant difference between WT and AIF Tg mice after correction for the loading control (*n* = 5/group).

Caspase-dependent apoptotic cell death was investigated by immunostaining of active caspase-3 in different brain regions at 24 h after HI, which is when caspase-3 activation reaches its peak^13^ (Fig. 5a). In the observed brain regions, caspase-3-positive cells were significantly increased in the cortex and striatum from AIF Tg mice as compared to WT mice, but no significant changes were seen in CA1 or the habenular nuclei (Fig. 5b–e). Quantification of active caspase-3-positive cells showed 1.6 times more cells in the cortex (*p* = 0.0481) and 1.2 times more cells in the striatum (*p* = 0.0012) from AIF Tg mice than in WT mice (Fig. 5c, e). Caspase-3 activity in the ipsilateral cortex was significantly increased, but no significant difference was found between WT and AIF Tg mice (Fig. 5f). Poly (ADP-ribose) polymerase-1 (PARP-1) was semi-quantified by immunoblotting at 24 h after HI. Compared with the contralateral (CL) hemisphere, cleaved PARP-1 was significantly increased in the ipsilateral (IL) hemisphere (*p* < 0.001), but no significant difference was found in the IL hemisphere between WT and AIF Tg mice (*p* = 0.7299) (Fig. 5g, h).

### AIF overexpression has no significant effects on mitochondria-related proteins after HI

**Fig. 6:**
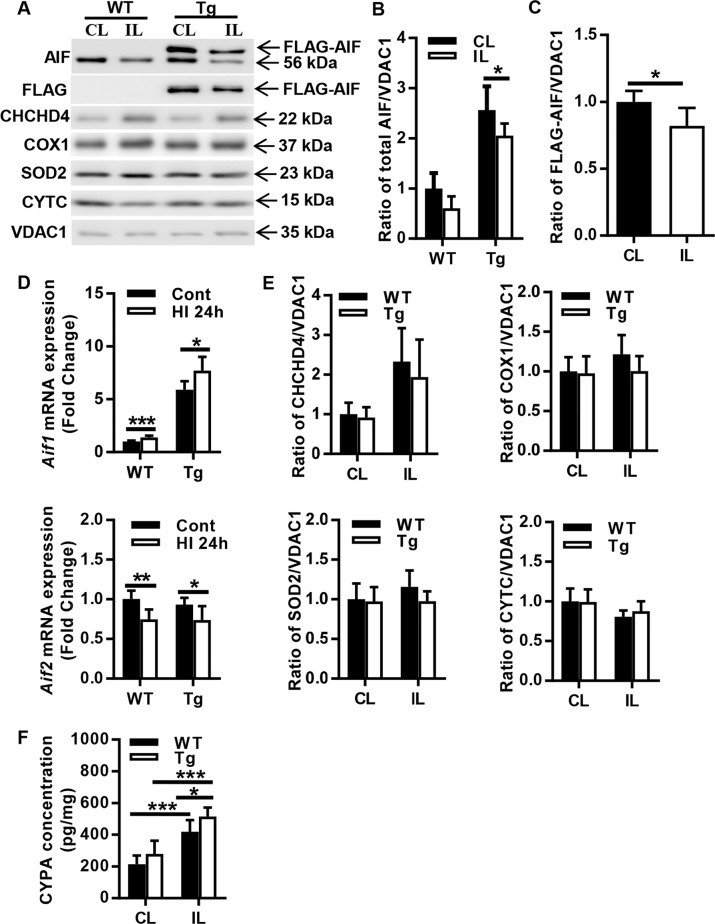
Changes in mitochondria-related proteins in the cortex after HI. **a** Representative immunoblotting of AIF, FLAG, CHCHD4, COX1, SOD2, and CYTC in the mitochondrial fraction of cortical tissue from the CL and IL hemispheres of WT and AIF Tg mice at 24 h after HI. **b** Quantification of AIF protein in the mitochondrial fraction from the CL and IL hemispheres of WT and AIF Tg mice at 24 h after HI (*n* = 6/group). A greater reduction in AIF from the mitochondria was seen in the IL of AIF Tg mice. **c** Quantification of FLAG-AIF protein in the mitochondrial fraction from the CL and IL hemispheres of AIF Tg mice at 24 h after HI (*n* = 6/group). FLAG-AIF was significantly released from the mitochondria in the IL of AIF Tg mice. **d** The mRNA expressions of *Aif1* and *Aif2* were determined by RT-qPCR in cortical tissue of WT and AIF Tg mice at 24 h post-HI. *Aif1* expression was increased in both WT and AIF Tg mice, but *Aif2* expression was decreased in both WT and AIF Tg mice (*n* = 6/group). **e** Quantification of mitochondria-related proteins (CHCHD4, COX1, SOD2, and CYTC) did not show any significant differences between WT and AIF Tg at 24 h after HI, but there was increased CHCHD4 expression in the IL of both WT and AIF Tg mice (*n* = 6/group). **f** CYPA in the nuclear fraction of cortical tissue was significantly increased in the IL hemisphere of both WT and AIF Tg mice at 24 h after HI. The increase was more obvious in AIF Tg mice (*n* = 6/group). *p < 0.05, **p < 0.01, ***p < 0.001.

To investigate the impact of AIF overexpression on mitochondrial cell death-related proteins after HI, the protein expression of total AIF, FLAG-AIF, CHCHD4, COX1, SOD2, and CYTC in the mitochondrial fraction of the cortical tissue was determined by immunoblotting at 24 h after HI (Fig. 6a). Comparing the CL hemisphere with the IL hemisphere, AIF protein was reduced in the IL hemisphere in both WT and AIF Tg mice, and this reduction was more pronounced in AIF Tg mice (*p* = 0.0284) (Fig. 6b), which indicates that more AIF was released from mitochondria after HI in the AIF Tg mice. Approximately 18% transgene-encoded (FLAG-positive) AIF was released from mitochondria at 24 h after HI (*p* = 0.019) (Fig. 6c). Because there are no specific antibodies to identify the two AIF isoforms, we used RT-qPCR to measure the mRNA expression of *Aif1* and *Aif2* at 24 h after HI. *Aif1* mRNA expression was significantly upregulated in both WT and AIF Tg mice at 24 h after HI compared to their controls (*p* < 0.001 for WT mice and *p* = 0.0160 for AIF Tg mice). In sharp contrast, *Aif2* was significantly downregulated in both WT and AIF Tg mice (*p* = 0.0031 for WT mice and *p* = 0.0352 for AIF Tg mice) (Fig. 6d). Quantification of mitochondria-related proteins CHCHD4 (*p* = 0.5403), COX1 (*p* = 0.1819), SOD2 (*p* = 0.1902), and CYTC (*p* = 0.6159) did not reveal any significant differences between WT and AIF Tg mice at 24 h after HI. However, CHCHD4 expression was increased in the IL hemisphere after HI in both WT and AIF Tg mice (Fig. 6e). Cyclophilin A (CYPA), an immunophilin, has a variety of intracellular functions, including signaling, protein trafficking, and the regulation of other proteins. CYPA participates in the nuclear translocation of AIF in neurons after cerebral hypoxia-ischemia^33^. The CYPA concentration in the nuclear fraction was increased significantly in the IL hemispheres of both WT and AIF Tg mice (both *p* < 0.001), and the concentration of CYPA in the nuclear fraction of AIF Tg mice was significantly higher than that of WT mice (*p* = 0.0488) (Fig. 6f).

### AIF overexpression has no effects on mitochondrial dynamics

**Fig. 7:**
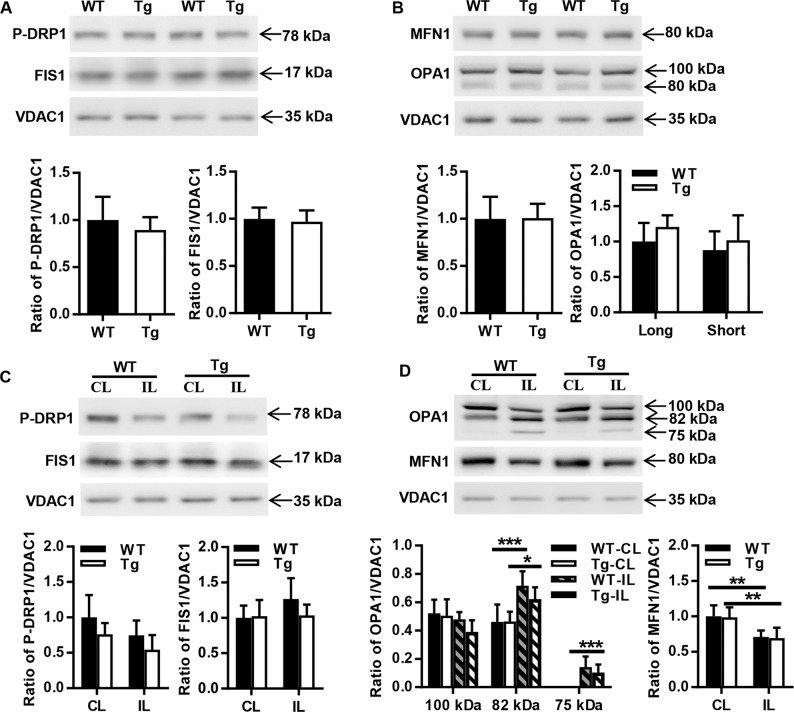
Effect of AIF overexpression on mitochondrial fission and fusion in the cortex. **a** Representative immunoblots of mitochondrial fission proteins (P-DRP1 and FIS1) in the mitochondrial fraction from cortical tissue of P9 WT and AIF Tg mice under physiological conditions. Quantification of P-DRP1 and FIS1 did not show a significant difference between WT and AIF Tg mice (*n* = 6/group). **b** Representative immunoblots of mitochondrial fusion proteins (OPA1 and MFN1) in the mitochondrial fraction from cortical tissue of P9 WT and AIF Tg mice under physiological conditions. Quantification of OPA1 and MFN1 did not show any significant difference between WT and AIF Tg mice (*n* = 6/group). **c** Immunoblotting of P-DRP1and FIS1 in the mitochondrial fraction from the cortical tissue of the CL and IL hemispheres in WT and AIF Tg mice at 24 h after HI. Quantification of P-DRP1 and FIS1 did not show any significant differences in the CL and IL hemispheres between WT and AIF Tg mice (*n* = 6/group). **d** Immunoblotting of OPA1 and MFN1 in the mitochondrial fraction from cortical tissue of the CL and IL hemispheres at 24 h after HI in WT and AIF Tg mice. The expression of MFN1 was significantly decreased at 24 h post-HI both in WT and AIF Tg mice, while the short form (82 kDa) and cleavage band of OPA1 were significantly increased at 24 h post-HI in both the WT and AIF Tg mice. Quantification of OPA1 and MFN1 did not show any significant differences in the CL and IL hemispheres between WT and AIF Tg mice (*n* = 6/group).

Mitochondrial morphology is determined by a dynamic equilibrium between organelle fusion and fission, and HI injury affects mitochondrial dynamics in the neonatal mouse brain^27^. To investigate the effect of AIF overexpression on mitochondrial dynamics, the expression of the fission and fusion-related proteins phospho-dynamin-related protein 1 (P-DRP1), mitochondrial fission 1 (FIS1), OPA1 mitochondrial dynamin like GTPase (OPA1), and mitofusin 1 (MFN1) were determined in both P9 WT and AIF Tg mice under physiological conditions. No significant changes were found between the two genotypes (*p* = 0.3854 for P-DRP1, *p* = 0.6708 for FIS1, *p* = 0.1313 for the long form of OPA1, *p* = 0.4922 for the short form of OPA1, and *p* = 0.9470 for MFN1) (Fig. 7a, b). The expression of the fission proteins P-DRP1 and FIS1 were further determined at 24 h after HI (Fig. 7c). The abundance of P-DRP1 was reduced in the IL hemisphere in both WT and AIF Tg mice, but this difference was not significant. The long form of OPA1 was reduced in both WT and AIF Tg mice at 24 h after HI, and it was much less abundant in AIF Tg mice. In contrast, the short form and the cleavage band of OPA1 were significantly increased at 24 h after HI in the two genotypes (*p* = 0.0003 for WT and *p* = 0.0206 for AIF Tg). Comparing the CL and IL hemispheres, MFN1 expression was also significantly decreased in both WT and AIF Tg mice at 24 h after HI (*p* = 0.0029 for WT and *p* = 0.0025 for AIF Tg). The increased short form and cleavage product of OPA1 along with the reduction of MFN1 indicate that mitochondrial fusion was compromised after HI injury. However, there was no difference between WT and AIF Tg mice with respect to the cleavage of OPA1 and the depletion of MFN1 (Fig. 7d).

## Discussion

AIF is a mitochondrial oxidoreductase that, if present in mitochondria, functions as an anti-apoptotic oxidoreductase^34^. AIF also regulates the assembly and maintenance of the respiratory complexes, thus influencing metabolic pathways and epigenetic processes^35^. Deficiency in AIF leads to severe mitochondrial dysfunction, causing muscle atrophy and neurodegeneration in model organisms as well as in humans^36–38^. The Harlequin mouse with a mutation derived from a proviral insertion in the X-linked *Aifm1* locus has an 80% reduction in *Aifm1* mRNA expression. As a consequence of AIF deficiency, neurons from adult Harlequin mice suffer from greater oxidative stress, enhanced cell cycle re-entry, and progressive cerebellar degeneration^39^. During apoptosis, AIF translocates from the mitochondria to the nucleus to act as a pro-apoptotic factor^40,41^. The pro-apoptotic activity of AIF appears to dominate over its neuroprotective function in neonatal mice, meaning that Harlequin mice manifest reduced neuronal cell loss in response to HI compared to normal WT controls^17^.

However, the effect of AIF overexpression has not yet been reported in vivo. The AIF Tg mice reported here exhibited 5.9 times higher *Aif1* mRNA expression (but no significant changes in *Aif2 mRNA*). In spite of the *Aif1* hyperexpression, we observed no change in the expression of mitochondrial cell death-related proteins or mitochondrial biogenesis-related proteins under physiological conditions. Comparing body weight gain and mortality with WT mice, no specific phenotypes were observed in the P9 AIF Tg mice, but the increasing numbers of DCX-positive cells in the granular layer of the dentate gyrus in 1-year-old AIF Tg mice indicated that excessive AIF might increase neuronal proliferation in the long term but not affect the early stages of brain development. The transcriptome analysis showed fewer than 2% DEGs in the P9 mouse brain, even when using a relatively loose criterion for the definition of DEGs. All of these results suggest that excessive AIF does not have any major impact on physiological functions.

Apoptosis is critical for central nervous system development in the immature brain, and many of the proteins involved in apoptosis are upregulated during brain development^10^. Our previous studies showed that AIF translocates to the nucleus in the neonatal brain after HI^28^ and that downregulation of AIF expression makes mice less vulnerable to HI injury^17^. Furthermore, blocking AIF translocation or downregulating AIF partner proteins reduces neuronal cell death and brain injury^15,18,33,42^, suggesting that AIF plays a causal role in cell death. In the AIF Tg mice, the AIF protein was more than two-fold increased and AIF nuclear translocation was much more pronounced and resulted in more severe brain injury after HI as compared to WT mice with normal AIF protein expression. More CYPA was found in the nucleus of AIF Tg mice indicating that this cytosolic protein participates in the nuclear translocation of excessive AIF. These results are in accordance with our previous findings in which reduced AIF or blocking AIF translocation leads to reduced injury, which indicates that AIF protein expression is positively correlated with brain injury after HI^17^. In contrast, selective knockout of exon 2 (which abolishes expression of AIF2 but not AIF1) aggravates cerebral injury after HI injury^32^, indicating that AIF1 and AIF2 differ in their role in the neonatal brain after HI.

Caspases have proteolytic activity and are able to cleave proteins at aspartic acid residues. Caspase-3 is the most important executioner caspase. In the AIF Tg neonatal mouse brain, a significant increase in active-caspase3-positive cells was only found in the cortex and striatum regions at 24 h after HI, but not in the CA1 or the habenular nuclei, which are different in the composition of neuronal subpopulations^43^. The hippocampus plays a role in memory consolidation and spatial navigation. The cell types are different between the hippocampus and the other brain regions, and the neuron densities in the main part of the hippocampus are lower than in the cortex^44^. The differences in populations and types might cause cells to undergo different cell death pathways in different brain regions^45^. Our present results indicate that the caspase3-independent cell death pathway is more pronounced than the caspase3-dependent pathway in the hippocampus.

AIF is critical for PARP-1-dependent cell death (parthanatos)^46–48^. Excessive activation of PARP-1 leads to the formation of poly(ADP-ribose) polymer that translocates from the nucleus to the mitochondria resulting in the release of AIF and accompanying over consumption of NAD+ that results in a decrease in ATP production^49^. PARP-1 is one of the caspase substrates, and cleavage of PARP-1 by caspases is considered to be a hallmark of apoptosis^50^. In this study, HI insult induced caspase-3 activation and PARP-1 cleavage at 24 h, but no significant differences were found between the WT and Tg group. As we know, different cell death pathways might be activated by common upstream initiators and might even share intersecting signal transduction cascades in a complex crosstalk. However, the specific modalities of such a crosstalk and the preference for one or the other (caspase-dependent, caspase-independent) pathway might differ in distinct brain regions, or even in a sex-biased fashion, depending on the pathological conditions^51–53^. This suggests that multipronged targeting of several cell death effectors is likely to be more effective in inhibiting cell death than acting on a single molecule only.

In living cells, mitochondria are continuously remodeled by fission and fusion events. These antagonistic processes link mitochondrial dynamics with the balance between energy demand and nutrient supply^54^. Highly fused mitochondria are efficiently formed under nutrient deprivation or upon exposure to certain forms of stress in order to optimize mitochondrial function and hence to maximize ATP synthesis. In contrast, mitochondrial fission is frequently observed in excess nutrient environments, such as in obesity. These dynamic changes mean that maintaining or promoting mitochondrial fusion or inhibiting mitochondrial fission might have a protective effect against HI-induced injury. AIF deficiency reportedly can disrupt mitochondrial dynamics^22^. However, in the current study, AIF overexpression did not have any influence on the expression of mitochondrial fusion or fission-related proteins under physiological conditions. HI injury might reduce mitochondrial fusion, as indicated by decreased expression of the fusion proteins MFN1 and the long form of OPA1, and might increase mitochondrial fission, as indicated by downregulation of P-DRP1. Indeed, published results suggest that HI reduces mitochondrial fusion and increases mitochondrial fission to aggravate neonatal brain injury^23,24,55,56^. However, no significant differences in proteins regulating mitochondrial fusion or fission were found between the WT and AIF Tg mice after HI, suggesting that the aggravation of HI injury in the neonatal brain due to excessive AIF expression is unlikely to be related to changes in mitochondrial dynamics.

There are some limitations in the current study. First, due to the fact that the mouse genotypes are complicated, we are restricted in the number of mice obtained from each of the genotypes, which prevented us from obtaining samples from more time points, such as 6 or 12 h post-HI. Second, even though some long-term study data in the non-injured mice have been included, we do not have long-term neurobehavioral function data after the HI injury. We found that AIF over expression aggravated brain injury after HI, but this would be more convincing if a long-term behavioral analysis could be performed.

In summary, our study demonstrates that excessive AIF does not initiate obvious phenotypic changes or changes to any physiological functions. Excessive AIF protein expression does, however, aggravate HI-induced injury mainly in the neonatal brain, correlating with a more prominent translocation of AIF release from the mitochondria to the nucleus of brain cells, as well as a brain region-specific increase in caspase-3 activation. Altogether, this study indicates a major role for AIF in neonatal HI brain injury. Future research should focus on strategies to prevent AIF release from the mitochondria and subsequent nuclear translocation after HI insult.

## Materials and methods

### Animals

The AIF overexpression transgenic mouse (AIF-Tg^flox/flox^) was constructed in a C57/Bl6 background using Rosa26 locus gene targeting, and AIF overexpression was regulated by Cre recombinase. The floxed mice were crossed with beta-actin-cre mice. The AIF-Tg^flox/+^-actin-cre (AIF overexpression, AIF Tg) and AIF-Tg^+/+^-actin-cre (wild type, WT) mice with reasonable body weight (4.0–5.5 g) at P9 and each litter with 5–8 pups were used in this study. A total of 15 pups were excluded because of death during surgery or during hypoxia, and a total of 114 mouse pups were used for analysis. No statistical methods were engaged to predetermine sample size, and instead we based our experimental design on numbers reported in previous studies^11,18^. All pups were grouped by genotypes, which means AIF Tg mice belong to experimental group and WT mice belong to control group. All the experimental protocol approved. All experimental procedures conformed to guidelines established by the Swedish Board of Agriculture (SJVFS 2019: 10), were approved by the Gothenburg Animal Ethics Committee (112/2014). The animal experiments were performed in the Laboratory for Experimental Biomedicine of Gothenburg University, and are reported in compliance with the ARRIVE (Animal Research: Reporting in vivo Experiments) guidelines. The sample collection time is indicated in Fig. 1a. Genomic DNA was isolated from tail samples (Qiagen, Hilden, Germany; 69506), and PCR was performed on a Biometra T3 Thermocycler (Biometra GmbH, Göttingen, Germany). The primers for the AIF transgenic flox gene were 5′-GAGTTCTCTGCTGCCTCCTG-3′ (forward), 5′-AAGACCGCGAAGAGTTTGTC-3′ (reverse for flox band, 215 bp), and 5′-CGAGGCGGATACAAGCAATA-3′ (reverse for wild type band, 322 bp), and the primer pair for the beta-actin-cre gene was 5′-CTGCCACGACCAAGTGACAGCAATG-3′ (forward) and 5′-GCCTTCTCTACACCTGCGGTGCTAA-3′ (reverse) to produce an amplicon of 326 bp. A 1.5% agarose gel electrophoresis system (Bio-Rad, California, US) and LAS 3000 cooled CCD camera (Fujifilm, Tokyo, Japan) were used for genotype detection.

### Cerebral HI

Postnatal day (P)9 mice of both sexes were anesthetized with isoflurane (5% for induction, 1.5–2.0% for maintenance) in a 1:1 mixture of air and oxygen, and the duration of anesthesia and surgery was <5 min. The right common carotid artery was cut between double ligatures. After the surgical procedure, the wounds were infiltrated with xylocaine. Pups were returned to their dams for 1 h and then placed in a chamber perfused with a humidified gas mixture (10% ± 0.01% oxygen in nitrogen) for 40 min at 36 °C. After the hypoxic exposure, the pups were returned to their dams until sacrifice. Control pups were subjected to all procedures except HI.

### Sample preparation for immunohistochemistry

Animals were deeply anesthetized with an overdose of sodium pentobarbital and perfused intracardially with PBS and 5% buffered formaldehyde (Histofix; Histolab, Gothenburg, Sweden). The brains were fixed in 5% buffered formaldehyde at 4 °C overnight. After dehydration with graded ethanol, the brains were embedded in paraffin and cut into 5-µm thick coronal sections, which were deparaffinized in xylene and rehydrated in graded ethanol. Antigen retrieval was performed by heating the sections in 10 mM boiling sodium citrate buffer (pH 6.0) for 10 min. The primary antibodies were as follows and were incubated with the sections overnight at 4 °C: mouse anti-MAP2 (1:1000 dilution, clone HM-2, Sigma, M4403), mouse anti-MBP (1:500 dilution, clone SMI94, BioLegend, 836504), rabbit cleaved caspase-3 (1:200 dilution, Asp175, Cell Signaling, 9661), rabbit anti-AIF (1:500 dilution, E20, Abcam, ab32516), and goat anti-DCX (1:500 dilution, C-18, Santa Cruz, sc-8066). After washing, the appropriate biotinylated secondary antibodies (1:200 dilutions; all from Vector Laboratories, Burlingame, CA, USA) were added to each section for 60 min at room temperature. The sections were visualized with a Vectastain Elite ABC Horseradish Peroxidase (HRP) Kit (Vector Laboratories, PK-6100).

For the immunofluorescence staining, the mixed primary antibodies of rabbit anti-AIF (1:500 dilution, E20, Abcam, ab32516) and mouse ANTI-FLAG (1:500 dilution, M2, Sigma, F1804) were incubated with the sections overnight at 4 °C. After washing, the mixed secondary antibodies of donkey anti-rabbit Alexa Fluor488 (1:500 dilution, Life Technology, A21206) and donkey anti-mouse Alexa Fluor555 (1:500 dilution, Life Technology, A31570) were added to each section for 120 min at room temperature. After washing, the sections were mounted on coverslips with ProLong^TM^ Gold anti-fade reagent with DAPI (Invitrogen, P36931).

### Brain injury evaluation

Brain injury was evaluated based on MAP2 and MBP immunostainings. Both hemispheres of each section were measured using Micro Image (Olympus, Japan). MAP2-positive and negative and MBP-positive tissue volume calculations as well as neuropathological scores of the gray matter from different brain regions were assessed as described previously^11^. The infarction volume was equal to the MAP2-negative volume in the IL hemisphere. MBP tissue loss ratio was calculated as: ((CL hemisphere − IL hemisphere)/CL hemisphere) × 100%. All evaluations were carried out by investigators blinded to group assignment.

### Cell counting

Area contours with fixed location were drawn and measured in every 50th section. The active caspase-3-positive cells and AIF-positive nuclei were counted within a defined area (one visual field) of the cortex (100×), striatum (200×), CA1 (200×), and habenular nuclei (200×). The DCX-positive cells in the granular layer of the dentate gyrus in 1-year-old mice were counted at 100× magnification. Three sections were counted from each brain with an interval of 250 μm. All of the counting was carried out by investigators blinded to group assignment.

### Sample preparation for immunoblotting and ELISA assay

The pups were sacrificed by decapitation at 24 h after HI. Tissue from the parietal cortex (including the hippocampus) in both hemispheres was rapidly dissected out and homogenized immediately on ice using a 2 ml Dounce tissue grinder set (Sigma, D8938), and isolation buffer was added (15 mM Tris-HCl, pH 7.6, 320 mM sucrose, 1 mM dithiothreitol, 1 mM MgCl_2_, 3 mM EDTA-K, and 0.5% protease inhibitor cocktail (Sigma, P8340)). Half of the homogenate was aliquoted and stored at −80 °C, and the other half was centrifuged at 800 × *g* for 10 min at 4 °C. The pellet fraction was washed with the isolation buffer, re-centrifuged with the same procedure, and saved as the nuclear fraction. The supernatant was further centrifuged at 9200 × *g* for 15 min at 4 °C, producing enriched mitochondrial fractions in the pellet and crude cytosolic fractions in the supernatant. The pellet was washed and centrifuged again and then resuspended with isolation buffer. All fractions were kept at −80 °C.

### Immunoblotting

Protein concentration was determined using the bicinchoninic acid method. Samples (65 µl) were mixed with 25 µl NuPAGE LDS 4× sample buffer (ThermoFisher Scientific, NP0007) and 10 µl reducing agent (ThermoFisher Scientific, NP0004) and heated at 70 °C for 10 min. Samples were run on 4–12% NuPAGE Bis-Tris gels (Invitrogen) and transferred to reinforced nitrocellulose membranes (Bio-Rad). After blocking with 5% fat-free milk in TBST buffer (20 mM Tris, 150 mM NaCl, and 0.1% Tween 20, pH 7.6) for 60 min at room temperature, the membranes were incubated with the following primary antibodies: rabbit anti-AIF (1:1000 dilution, E20, Abcam, ab32516), mouse ANTI-FLAG (1:1000 dilution, M2, Sigma, F1804), mouse anti-CHCHD4 (1:200 dilution, C-12, Santa Cruz, sc-365137), rabbit anti-COX1 (1:1000 dilution, EPR19628, Abcam, ab203912), rabbit anti-PGC1α (1:1000 dilution, ThermoFisher, PA5-38021), rabbit anti-TFAM (1:1000 dilution, ABclonal, A13552), rabbit anti-SOD2 (1:1000 dilution, ABclonal, A1340), mouse anti-cytochrome c (1:500 dilution, 6H2, Santa Cruz, sc-13561), rabbit anti-phospho-DRP1 (1:1000 dilution, Ser637, Cell Signaling, 4867), mouse anti-OPA1 (1:1000 dilution, BD bioscience, 612606), mouse anti-MFN1 (1:500 dilution, 11E9-1H12, Novus Biologicals, NBP1-71775), rabbit anti-FIS1 (1:500 dilution, FL-152, Santa Cruz, sc-98900), rabbit anti-PARP-1 (1:1000 dilution, E102, Abcam, ab32138), rabbit anti-cleaved PARP-1 (1:1000 dilution, E51, Abcam, ab32064), goat anti-Lamin B (1:200 dilution, M-20, Santa Cruz, sc-6217), and mouse anti-VDAC1 (1:500 dilution, B-6, Santa Cruz, sc-390996) overnight. After washing, the membranes were incubated with peroxidase-labeled goat anti-rabbit IgG antibody (1:2000 dilution, Vector, PI-1000) or peroxidase-labeled horse anti-mouse IgG antibody (1:4000 dilution, Vector, PI-2000) or horse anti-goat IgG antibody (1:2000 dilution, Vector, PI-9500). Immunoreactive species were visualized using the SuperSignal West Pico PLUS Chemiluminescent Substrate (ThermoFisher Scientific, 34580) and LAS 3000 cooled CCD camera (Fujifilm, Japan).

### OXPHOS complex I enzyme activity assay

The mitochondrial fraction was used for the complex I enzyme activity assay (Abcam, ab109721). Samples were prepared by following the manufacture’s instruction and were diluted to the desired concentration in incubation solution. The sample (200 µl) was added to the pre-coated microplate (200 µl of incubation solution was added to measure the background signal), and the microplate was incubated for 3 h at 21 °C. After washing, 200 µl of assay solution (1× dilution buffer, 20× NADH, and 100× dye) was added to each well. The plate was read in kinetic mode, and the OD at 450 nm was measured at 21 °C. The result was expressed as mOD/min/100 µg protein.

### Cyclophilin A measurement

The nuclear fraction was used for CYPA measurement (Abbexa, abx585050) following the manufacturer’s instructions. The sample and standard solution were added to a pre-coated plate and incubated for 90 min at 37 °C. After the incubation, the biotin-conjugated antibody was added. After incubation at 37 °C, 100 µl HRP solution was added and incubated for 30 min at 37 °C. After washing, 90 µl 3,3′,5,5′-Tetramethylbenzidine substrate was added and incubated for 20 min at 37 °C. After the final incubation, sulfuric acid stop solution was added and the absorbance at 450 nm was immediately measured. The result was expressed as pg/mg protein.

### Caspase-3 activity assay

A total of 25 μl homogenate was mixed with 75 μl extraction buffer containing 50 mM Tris-HCl (pH 7.3), 100 mM NaCl, 5 mM EDTA, 1 mM EGTA, 1 mM PMSF, and 1% protease inhibitor cocktail on a microtiter plate. After incubation for 15 min at room temperature, 25 μM caspase-3-substrate (Ac-DEVD-AMC, Peptide Institute, 670613) in 100 μl assay buffer was added. Caspase-3 activity was measured using a Spectramax Gemini microplate fluorometer (excitation/emission wavelength 380/460 nm every 2 min for 2 h at 37 °C) and expressed as pmol AMC/mg protein per minute^28^.

### RNA extraction and sequencing

Cortical samples from P9 WT and AIF Tg mice were prepared for RNA sequencing. Total RNA from each sample was extracted using a RNeasy Mini kit (Qiagen, 74104), and the library preparation was done using an MGI Easy™ mRNA Library Prep Kit (BGI, Wuhan, China) following the manufacturer’s instructions. The sequencing library was used for cluster generation and sequencing on the BGISEQ-500 system (BGI)^57^. The sequencing was repeated ten times, and the DESeq method was used to screen for between the two groups according to the criterion of *p* < 0.05. GO term classification was performed using the cluster Profiler R package^58^.

### RT-qPCR

Total RNA concentration and purity were determined using a Nanodrop spectrophotometer (Nanodrop Technologies, Wilmington, USA). One microgram of total RNA was reverse transcribed using the QuantiTect Reverse Transcription kit (Qiagen, 205311). RT-qPCR was performed using the LightCycler 480 instrument (Roche Diagnostics, Mannheim, Germany) and the SYBR green (ThermoFisher Scientific, 0253) technique according to the manufacturer’s instructions. The primers used in the qPCR reactions were designed by Beacon Designer software (PREMIER Biosoft) and were as follows: general *Aif* (sense: 5′-TATTTCCAGCCACCTTCTTTC-3′, anti-sense: 5′-TTCACCATGTTGCCTCTTAC-3′), *Aif1* (sense: 5′-AGTCCTTATTGTGGGCTTATC-3′, anti-sense: 5′-GCAATGGCTCTTCTCTGTT-3′), *Aif2* (sense: 5′-TTCTTAATTGTAGGAGCAACAGT-3′, anti-sense: 5′-CCCATCACTCTTTCATTGTAT CT-3′), and *Sdha* (the reference gene) (sense: 5′-TTGCCTTGCCAGGACTTA-3′, antisense: 5′-CACCTTGACTGTTGATGAGAAT-3′). The relative expression levels of mRNAs were calculated according to the formula of 2^−(ΔΔCT)^.

### Statistical analysis

GraphPad Prism 6 Software (GraphPad Software, San Diego, CA, USA) was used for all analyses. Comparisons between groups were performed by Student’s *t*-test, and data with unequal variance were compared with the Mann–Whitney *U*-test. Two-way ANOVA followed by Sidak’s post hoc test was used for multiple comparisons of data from more than two groups. Results are presented as means ± standard deviation, and *p* < 0.05 was considered statistically significant.
